# Cone-Beam Computed Tomography-Guided Online Adaptive Radiotherapy: Promising Results for Bladder Cancer Case

**DOI:** 10.7759/cureus.68863

**Published:** 2024-09-07

**Authors:** Sana Azzarouali, Karin Goudschaal, Jorrit Visser, Arjan Bel, Laurien Daniëls, Duncan den Boer

**Affiliations:** 1 Radiation Oncology, Amsterdam UMC location Vrije Universiteit Amsterdam, De Boelelaan 1117, Amsterdam, NLD; 2 Radiation Oncology, Amsterdam UMC location University of Amsterdam, Meibergdreef 9, Amsterdam, NLD

**Keywords:** adaptive radiotherapy, bladder cancer, cbct, cbct-guided, fiducial markers, focal boost, image-guided, online adaptive, radiotherapy

## Abstract

Bladder radiotherapy is challenging due to daily anatomical variations and unpredictable bladder filling, particularly affecting tumors in the cranial part. Conventional radiotherapy requires large planning target volume margins to manage these uncertainties, but this can expose healthy tissue to high radiation doses, increasing the risk of acute and late toxicity. Our aim was to study the potential to limit high-dose exposure to healthy tissue by comparing daily online adaptive radiotherapy (oART) with conventional, non-adaptive radiotherapy (non-ART).

The comparison was performed on a bladder cancer patient treated with a simultaneous integrated boost while having a challenging tumor location in the cranial part of the bladder. Liquid fiducial markers aided during the localization of the tumor bed to deliver this focal boost. The dose distribution of oART fractions performed in the clinic was compared with simulated non-ART fractions on the post-treatment cone-beam computed tomography (CBCT).

The results showed that while maintaining target coverage of the bladder and gross tumor volume in 100% of the fractions for both workflows, the high dose exposure to organs-at-risk was lower for oART. The small bowel received statistically significantly (p ≤ 0.05) less dose with oART compared to non-ART, with a median volume difference of 20 cm^3^ receiving 95% of the prescribed dose (55 Gy). The total volume of tissue outside the target receiving 95% of the prescribed dose was also smaller for oART compared to non-ART (p ≤ 0.05). The follow-up of two years showed that the patient had no long-term toxicity effects.

Therefore, CBCT-guided oART has been shown to offer a conformal treatment for a challenging patient and can provide a clear advantage in the treatment of bladder cancer.

## Introduction

For the treatment of muscle-invasive bladder cancer, radical cystectomy has long been considered the standard but can have significant side effects [1]. Radiotherapy treatment (RT) combined with transurethral resection of bladder tumor (TURBT) and chemotherapy has been demonstrated to be an effective alternative with the advantage of organ preservation [2,3]. However, a challenge of RT is the interfraction and intrafraction uncertainties mainly caused by daily variations in the exact shape, location, and size of the targets and variable bladder filling, respectively. With conventional image-guided radiotherapy, the reference plan is used for each fraction by first acquiring a daily image to position the patient, after which the treatment plan is delivered. Large safety margins (extracted from population-based studies) are needed to account for uncertainties both between (interfraction) and within (intrafraction) treatment sessions [4,5]. Including a simultaneous integrated boost (SIB) to RT, in which a higher dose is given to the tumor bed and a lower dose to the bladder (and pelvic lymph nodes), allows for reduced toxicity [2,6]. Several studies have shown that most variations (caused by bladder filling) are present in the cranial and ventral part of the bladder, making it more challenging to give this SIB to a patient with a tumor bed in this region [7,8].

Furthermore, considering the small bowel being close to the tumor receiving this boost dose, a conformal treatment is needed that can deal with the daily variations to reduce the amount of high dose received by the small bowel. Online adaptive radiotherapy (oART) aims to limit the interfraction variations by using the daily image to create a new structure set based on the anatomy of that moment and reoptimize the treatment plan, allowing for smaller margins [9]. This results in less dose to organs-at-risk (OAR) and other healthy tissue surrounding the bladder [10-12]. Therefore, oART offers the possibility to deliver a focal boost to the tumor (bed) that shows large day-to-day variations in position and shape. This reduces the dose received by the healthy part of the bladder wall and limits the chance of toxicity effects. Our previous study and the study of Åström et al. have demonstrated the feasibility of the oART workflow for bladder cancer [10,12]. Most studies have performed the evaluation on the daily image; however, including the intrafraction variation by evaluating the post-treatment image is necessary but currently lacking. Our aim was to evaluate both a conventional, non-adaptive RT workflow (non-ART) and an oART workflow for a patient in which the tumor was located at the cranial part of the bladder. The analysis was done by comparing a cone-beam computed tomography (CBCT)-guided oART workflow performed in the clinic with a non-ART workflow simulated on the same patient. A dosimetric analysis was done, evaluating the clinical goals and constraints, on the post-treatment CBCT.

## Case presentation

Patient data

**Figure 1:**
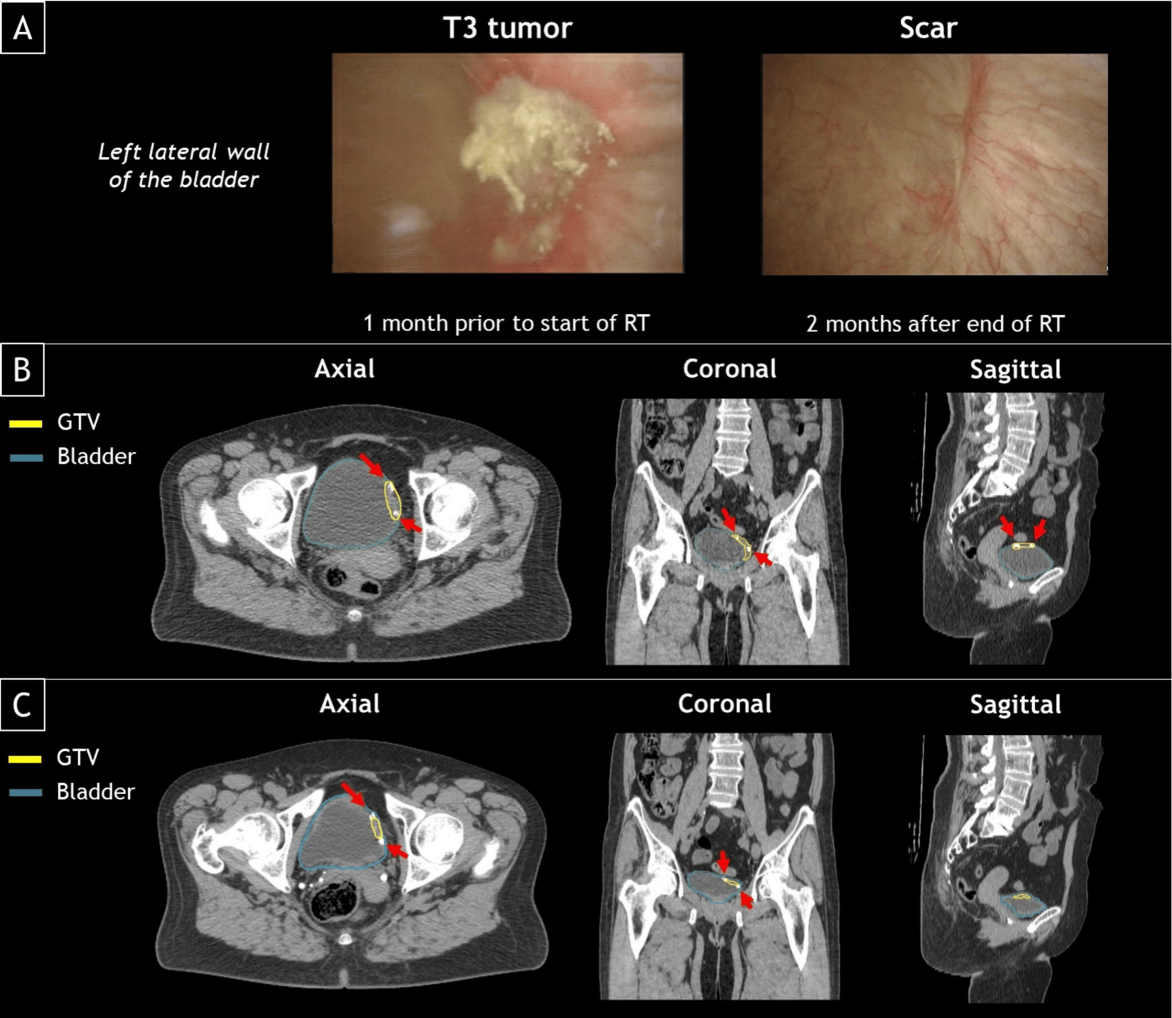
Cystoscopy and pretreatment CTs Images from the cystoscopy before and after radiotherapy treatment (RT) (A). Two CTs were acquired at 15-minute time intervals during pretreatment for manual contouring and to estimate the amount of intrafraction bladder filling. The CT in which the bladder was more filled (B) was used to make a reference treatment plan for the online adaptive radiotherapy (oART) workflow, and the other CT (C) was used for the non-adaptive radiotherapy (ART) simulated workflow. Fiducial markers (white blobs) aided the contour delineation process of the gross tumor volume (GTV) (see arrows).

A 60-year-old female patient presented with irritative micturition complaints without hematuria. During cystoscopy, a tumor was observed on the left lateral wall of the bladder (Figure 1A). After the initial biopsy, a transurethral resection of the tumor was performed. Pathology showed a muscle-invasive high-grade urothelial carcinoma without carcinoma in situ. A CT intravenous pyelogram scan showed no abnormalities in the upper urinary tract, no hydronephrosis, and no dilation of the urinary tract. There was a suspicion of a T3 bladder tumor. A CT of the thorax showed no pulmonary abnormalities. Positron emission tomography (PET)-CT scans revealed no indications of lymph nodes or distant metastases. The final diagnosis was a cT3N0M0 muscle-invasive high-grade urothelial carcinoma of the bladder.

The patient was counseled regarding the treatment options, which included cystectomy with lymph node dissection or bladder-sparing treatment with chemoradiotherapy. The patient opted for the latter and participated in the Chemoradiotherapy Combined With Immunotherapy in Muscle-Invasive Bladder Cancer (CRIMI) trial (NCT038442556). She received chemotherapy (one-time mitomycin-C (IV) and daily capecitabine (orally)) starting on the first day of RT. RT was given over a period of four weeks with a total of 20 fractions. The bladder and pelvic lymph nodes (internal iliac, obturator, hypogastric, and perivesical until the lower part of the sacroiliac joint) received a total dose of 40 Gy. A SIB dose of 15 Gy was given to the tumor bed (or any residual tumor).

Clinical workflow (oART)

Before obtaining a planned CT scan, liquid fiducial markers (BioXmark, Nanovi A/S, Kongens Lyngby, Denmark) were injected by a urologist at the edges of the tumor bed. These markers assist in defining the target (tumor bed) for both pretreatment CT and online fractions on CBCT in order to deliver a focal boost as a SIB. Fiducials were placed submucosally within a margin of 0-5 mm around the (remnant) tumor or the resection scar (referred to as “GTV,” i.e., gross tumor volume) using rigid cystoscopy. Four dots of each 0.1-0.2 cm³ in volume were placed. The patient was instructed to drink 0.3 L of water after emptying her bladder and then avoid drinking for 1.5 hours before acquiring two pretreatment CT scans (Discovery CT, GE Medical Systems, Milwaukee, WI). The CTs were performed with the patient in a supine position, with arms on the chest (including a thorax support for comfort) and knee support. The two CT scans (with 15-minute time intervals) were made to account for intrafraction bladder filling and determine patient-specific planning target volume (PTV) margins for the bladder (Figure 1). The second CT (Figure 1B), in which the bladder volume was larger, was used to manually delineate the targets: GTV, bladder, the urethra (1 cm proximal), and first pelvic lymph nodes (obturator, internal iliac, hypogastric, and external lymph nodes extending to the lower part of the sacroiliac joint). The OARs were also delineated and defined as the rectum, small bowel, bowel bag, sigmoid colon, and left and right femur heads. A 5 mm clinical target volume (CTV) margin was applied around the GTV (CTV_GTV_). A 5 mm PTV margin was used for CTV_GTV_. For the urethra and pelvic lymph nodes, a PTV margin of 7 and 5 mm was used, respectively. The patient-specific PTV margins for the bladder were 7 mm in all directions except for the cranial direction, which was 15 mm. A volumetric modulated arc therapy (VMAT) treatment plan was generated (three arcs, 6 MV flattening filter-free (FFF)) and used as a reference plan, which was made on the second CT with the most filled bladder (Ethos Therapy™ v1.1, Varian, a Siemens Healthineers Company, Palo Alto, CA). Before the online treatment, the patient received the same drink instructions as before the planning CT to aim for a filled bladder. The oART workflow (Ethos Therapy™ v1.1) consisted of acquiring a daily CBCT (CBCT1), delineation of the structure set, treatment plan optimization, acquiring a CBCT (CBCT2) for position verification (including a couch shift to maximize GTV coverage), RT delivery, and post-treatment imaging (CBCT3). An independent secondary dose calculation (Mobius, Varian, a Siemens Healthineers Company, Palo Alto, CA) was performed for plan quality assurance (for more details of the oART workflow, see Azzarouali et al. [12]).

Simulated workflow (non-ART)

**Figure 2:**
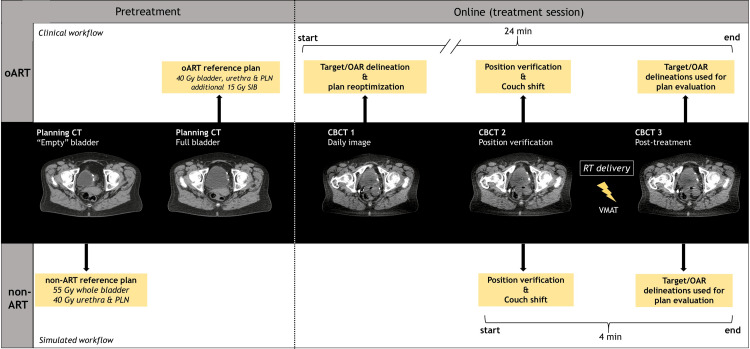
The (clinical) online adaptive radiotherapy and (simulated) non-adaptive radiotherapy workflow The reference plan of the online adaptive radiotherapy (oART) workflow was made in the treatment planning system of Ethos (Ethos Therapy™ v1.1, Varian, a Siemens Healthineers Company, Palo Alto, CA) based on a full bladder CT with 40 Gy to the bladder and pelvis lymph nodes (PLN) and an additional boost dose of 15 Gy to the tumor bed. For the non-ART workflow, this reference treatment plan was made in Eclipse (Eclipse Treatment Planning System v16.1, Varian, a Siemens Healthineers Company, Palo Alto, CA) based on an “empty" bladder CT with 55 Gy to the whole bladder and 40 Gy to the PLN. The imaging steps of the oART workflow consisted of cone-beam computed tomography (CBCT) acquisition. These same CBCTs were also used to simulate the non-ART workflow. The delineations from the post-CBCTs were used to evaluate both the daily reoptimized (adapted) plans for the oART workflow and the reference plan after performing a daily bone match for the non-ART workflow. The median on-couch time of each workflow is also given for this patient.

As mentioned in the previous section, to estimate the bladder filling speed, two CTs were made. The first (in which the bladder volume was lower) was used for this simulated non-ART workflow (Figure 1C). The bladder volume on this CT was 106 mL, which is lower than 126-136 mL and, therefore, falls in the “empty bladder” regime, as reported in the literature [4,7]. The applied PTV margins for this simulation were 11 mm (17 mm cranial) for the bladder, 5 mm for the pelvic lymph nodes, and 7 mm for the urethra, based on population-based margins reported in the literature [5,10,13]. To be in line with the most common practice, the total planned dose was 55 Gy for the whole bladder (including GTV) and 40 Gy for the pelvic lymph nodes and urethra [10,14-15]. A VMAT treatment plan (three arcs, 10 MV) was made (Eclipse Treatment Planning System v16.1, Varian, a Siemens Healthineers Company, Palo Alto, CA). A typical non-ART session consists of acquiring a daily image and performing a bone match between this daily image and the reference plan and RT delivery [10-12]. The same CBCTs that were used for position verification in the clinical oART workflow (i.e., CBCT2) right before the start of irradiation were used for the position verification for this non-ART simulation by means of a bone match with the CT of the reference plan (Velocity v4.1, Varian, a Siemens Healthineers Company, Palo Alto, CA). After initial alignment, visual inspection was done, and a subsequent soft tissue match was performed when required (for this specific patient, this was never the case). The non-ART and oART workflow are schematically illustrated in Figure 2.

Statistical analysis

An analysis of the dose distribution of each fraction was performed (Velocity v4.1) using manually delineated contours (semi-automatically delineated by adjusting the structure set proposed by the simulation environment of Ethos TherapyTM) from the post-treatment CBCT (CBCT3). The target coverage (V_95%_) and high dose exposure (V_52.25Gy_/V_38Gy_) to the OARs and tissue volume outside the targets were extracted. A paired Wilcoxon signed-rank test was performed to compare the treatment plans of the fractions from the oART and non-ART workflow. As the clinical drink instructions (used during oART) aim for a filled bladder, while a conventional treatment would require an empty bladder, fractions with a bladder volume exceeding 126 mL were excluded to come to a fairer comparison [4,7].

Dosimetric results

**Figure 3:**
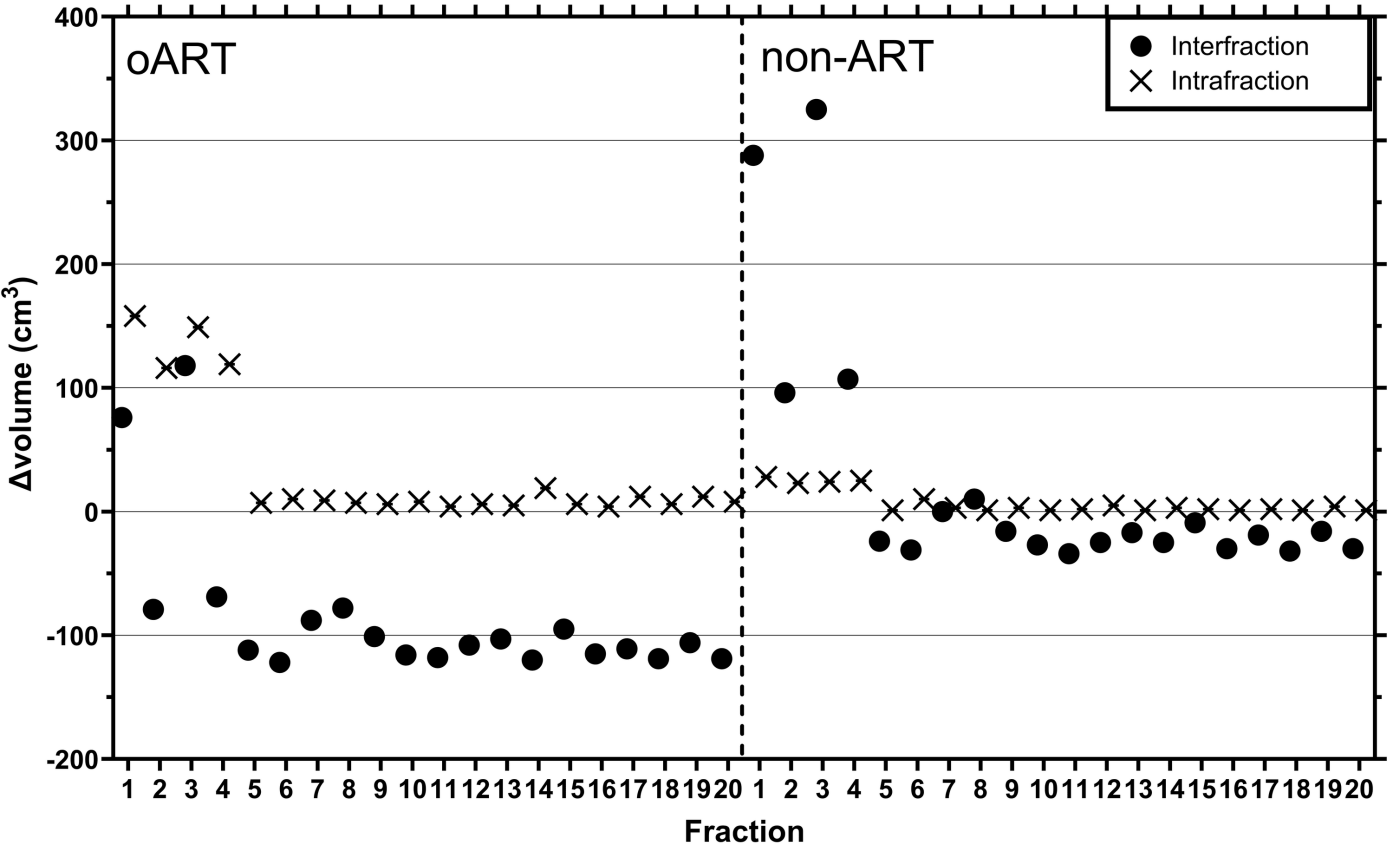
Interfraction and intrafraction variation in bladder volume during the non-adaptive radiotherapy and online adaptive radiotherapy workflow Interfraction (planning CT to daily cone-beam computed tomography (CBCT)) and intrafraction (daily CBCT to post-treatment CBCT) bladder volume differences were analyzed for each fraction of the online adaptive radiotherapy (oART) and non-adaptive radiotherapy (ART) workflows (n = 20). For the non-ART workflow, the CBCT used for position verification was considered the daily image.

During the clinical oART workflow, fractions one to four showed higher intrafraction bladder filling compared to the subsequent fractions (Figure 3). The bladder volume on the position verification CBCT of these fractions exceeded 126 mL. These fractions were, therefore, excluded from the evaluation (the remaining 16 fractions were included).

**Figure 4:**
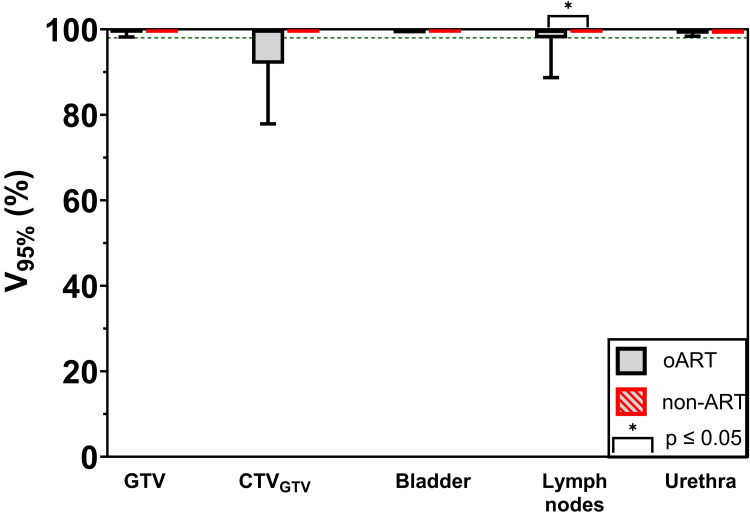
Target coverage with non-adaptive radiotherapy and online adaptive radiotherapy treatment plans evaluated on post-treatment cone-beam computed tomography The volume of the targets receiving a minimum of 95% of the prescribed dose (55 Gy or 40 Gy) with the online adaptive radiotherapy (oART) and non-adaptive radiotherapy (ART) treatment plan on the post-treatment CBCT (n = 16 fractions). The bladder was prescribed 55 Gy during non-ART and 40 Gy during oART. The dotted line illustrates the clinical requirement of 98%.

During all fractions, a reoptimized (adapted) treatment plan was selected, and all of them showed a passing rate of 100% for the gamma index (3%/3 mm) during the online independent dose calculation. Furthermore, all of the evaluated oART fractions met the clinical requirements for the target coverage of the GTV and the bladder on the post-treatment CBCT (Figure 4), which was also 100% for the non-ART workflow. There was no statistical (p ≤ 0.05) difference in target coverage between the two workflows except for the pelvic lymph nodes, which was higher for the non-ART workflow. Considering the CTV_GTV_, 75% and 100% of the fractions met the clinical requirement for target coverage for the oART and non-ART workflow, respectively.

**Figure 5:**
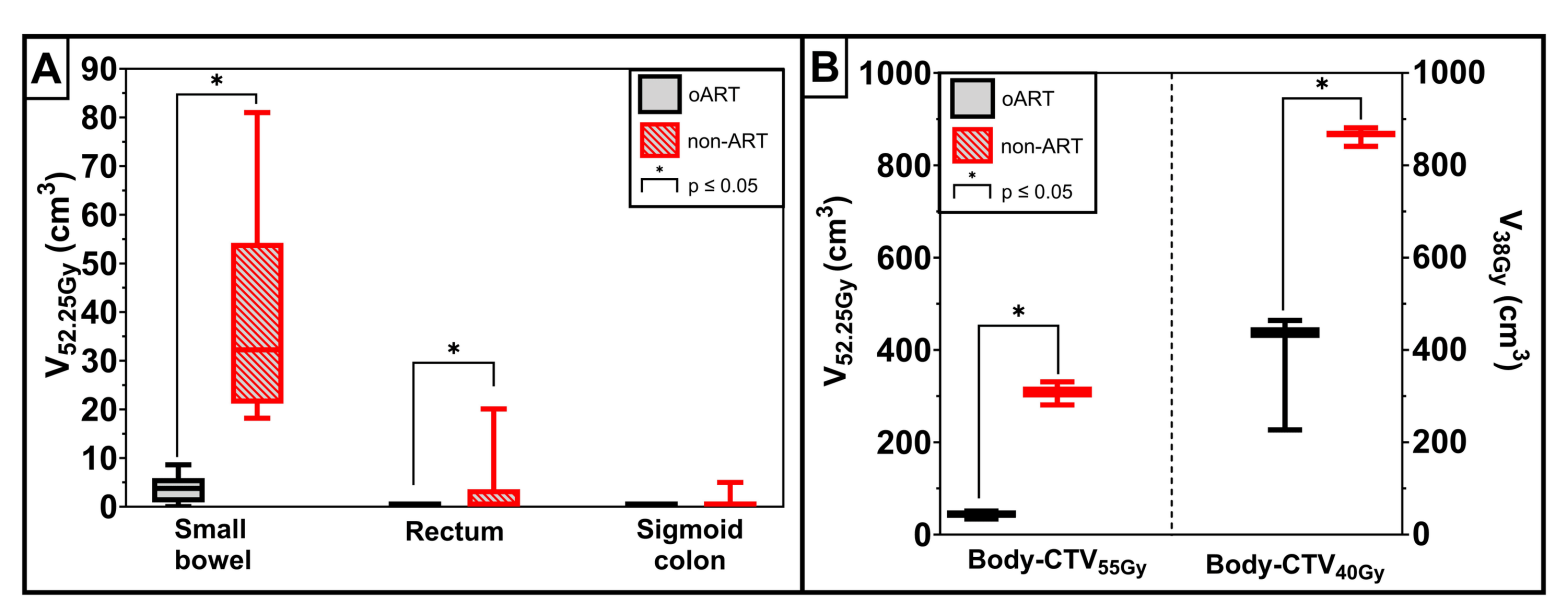
High-dose exposure to organs at risk and tissue volume outside the targets on post-treatment cone-beam computed tomography (A) Volume of the organs at risk (OARs) small bowel, rectum, and sigmoid colon receiving a minimum of 95% of 55 Gy (V_52.25Gy_) for the online adaptive radiotherapy (oART) and non-adaptive radiotherapy (ART) treatment plan on the post-treatment CBCT (n = 16 fractions). (B) The V_52.25Gy_ and V_38Gy_ (volume receiving a minimum of 95% of the prescribed dose) outside the targets prescribed with the same dose level (55 Gy or 40 Gy) for the oART and non-ART treatment plan on the post-treatment cone-beam computed tomography (CBCT) (n = 16 fractions), e.g., clinical target volume (CTV)_55Gy_ is defined as the combined CTV of all targets that were prescribed 55 Gy. Body-CTV_55Gy_ is defined as the tissue inside the body structure but outside the CTV_55Gy_.

**Figure 6:**
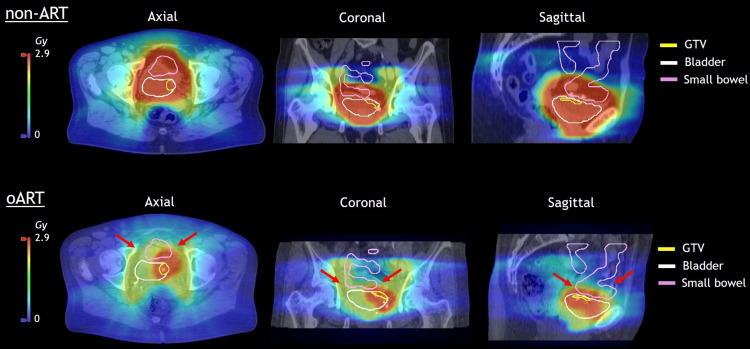
Dose exposure of the small bowel in the worst non-adaptive radiotherapy case compared to the online adaptive radiotherapy treatment plan Worst case fraction (fraction 11) of the non-adaptive radiotherapy (ART) workflow in which 81 cm^3^ of the small bowel would have received a high dose (52.25 Gy), while with online adaptive radiotherapy (oART), this volume reduced to 3 cm^3^ (pointed out by arrows). The reference CT and dose distribution are given for the non-ART treatment plan with the contours from the post-treatment cone-beam computed tomography (CBCT). The dose distribution of the reoptimized oART treatment plan is also given with these post-treatment CBCT contours and the daily CBCT.

With the reoptimized oART treatment plans, the volume of tissue outside the targets received significantly (p ≤ 0.05) less dose compared to the non-ART fractions (Figure 5). The small bowel and rectum also received statistically significantly less dose with the oART treatment plans than would have been received with the non-ART treatment plans (Figure 5A). The median difference in volume receiving 95% of the prescribed dose (55 Gy) was 29 cm^3^ (range: 12-78 cm^3^) for the small bowel. An example of a session in which the non-ART treatment plan would have resulted in a significant small bowel volume receiving a high dose is illustrated in Figure 6. With non-ART, 81 cm^3^ of the small bowel would have received a high dose (52.25 Gy) and reduced to 3 cm^3^ with oART.

Acute toxicity

Nausea and vomiting were observed due to capecitabine, but no diarrhea. Other observations were an increased frequency of urination (Common Terminology Criteria for Adverse Events (CTCAE) grade 1) and urinary tract pain (grade 1). Solifenacin was initiated with a good effect on the bladder symptoms. Skin toxicity was observed during immunotherapy as part of the CRIMI study. A general symptom was the patient experiencing fatigue.

Late toxicity and follow-up

For the follow-up, two years until now, a cystoscopy was performed two months after completing chemoradiation therapy. A scar was observed on the left lateral wall (Figure 1A), and there was no residual disease. Subsequent cystoscopies were performed every three months since the initial scope, with no local recurrence observed. A CT of the thorax/abdomen was conducted at six months and showed no evidence of locoregional or distant metastasis.

## Discussion

For the treatment of muscle-invasive bladder cancer, radical cystectomy has long been considered the standard despite its significant side effects, whereas radiotherapy offers an effective alternative with the advantage of organ preservation [1-3]. Therefore, non-ART uses large population-based PTV margins, while oART allows for smaller PTV margins through daily reoptimization. While most oART studies evaluate using the daily image, it is necessary to account for intrafraction variation by using post-treatment images, which is currently lacking. In our study, we compared oART with conventional non-ART for a patient with a challenging tumor bed position, as observed on post-treatment CBCT. The oART workflow demonstrated a reduction in high dose (52.25 Gy) to OARs and surrounding tissue outside the targets compared to the non-ART workflow. For the small bowel, this volume reduction was a median of 29 cm^3^ (range: 12-78 cm^3^), and if we consider all the tissue outside the CTV, this median volume reduction became 268 cm^3^ (range: 241-289 cm^3^)

The median on-couch time (daily image acquisition until the end of RT delivery) of a CBCT-guided oART session takes 22 minutes, which is longer than a typical non-ART session [10,12]. However, oART enabled smaller bladder PTV margins for this patient by mitigating interfraction uncertainties while maintaining statistically equivalent target coverage for the bladder compared to non-ART on post-treatment CBCT (100% met the clinical requirement).

For MR-guided oART, a typical session takes approximately 10 minutes longer than a CBCT-guided oART session, which can result in lower conformity [12,16-18]. This increase in duration is mainly caused by the time needed for the contour delineation process and the plan reoptimization, which eventually can allow for more intrafraction bladder filling.

Furthermore, using VMAT as a delivery method has also been shown to result in a more conformal treatment plan and fewer monitor units [19]. This leads to shorter delivery times and may help reduce intrafraction bladder filling during RT delivery. The implementation of CBCT-guided oART that we employed for this treatment supports the use of VMAT as the RT delivery method, whereas currently, the clinically available implementations of MR-guided oART techniques lack this capability. Previous studies indicate that the conformity index is lower for MR-guided oART compared to CBCT-guided oART, suggesting more doses to healthy tissue [10,12,16-17].

Regarding the GTV, the target coverage was also statistically similar between both workflows, and the clinical requirement was met in 100% of the fractions. Nevertheless, in the oART workflow, only the tumor bed received the prescribed 55 Gy, whereas, in the non-ART workflow, the entire bladder received this dose, increasing the chance of toxicity effects [2,6]. Another consideration to take into account is that this study focused on a special case patient with a complex anatomy, presenting significant challenges for the oART workflow with an SIB included. Therefore, extending this study to a larger patient population could yield even more promising outcomes.

Currently, the Adaptive Radiation Therapy of Bladder Cancer: An Individualized Approach (ARTIA-Vesica) trial (NCT05295992) is an ongoing prospective study designed to evaluate the effectiveness of oART in reducing the rates of acute gastrointestinal/genitourinary toxicity compared to non-ART in patients with muscle-invasive bladder cancer. This approach seeks to personalize RT by adjusting the treatment plan daily based on anatomical changes, potentially leading to better patient outcomes. Given the potential advantages of VMAT for bladder cancer patients, particularly its ability to deliver radiation more quickly, thereby reducing the impact of bladder filling during treatment, it would be valuable to consider the integration of VMAT into future trials.

Study limitations

A limitation of the non-ART simulation was the difference in bladder preparation protocols; the clinical oART workflow included a drink instruction to achieve a filled bladder online, whereas an empty bladder is typically preferred for non-ART. To address this, we selected fractions based on bladder volume observed in daily images used for position verification. Despite the drink instructions aimed at a filled bladder, there was still unpredictable variation in online bladder volume, consistent with existing literature [5,7-8].

CBCT2 was used as a starting point to simulate the non-ART workflow, and CBCT3 was used for the evaluation process with a realistic four-minute time interval representative of a clinical non-ART workflow. Alternatively, using CBCT1 as a starting point for the simulation and CBCT2 for the evaluation with a longer, for IGRT, unrealistic on-couch time of ~20 minutes would unjustly impact the non-ART results in a negative way. Instead, using CBCT1 as a starting point and using another CBCT made a few minutes later for the evaluation would have been a more ideal scenario to perform the simulation. Unfortunately, this CBCT was not available in the clinical workflow. However, the short on-couch time between CBCT2 and CBCT3 (Figure 2), in combination with the limited intrafractional bladder filling observed in this patient (Figure 3), still provides illustrative results regarding the consequences of a non-ART treatment for this patient.

Evaluating target coverage on post-treatment images tends to underestimate coverage because the bladder volume increases during fractions, and a higher dose is received at the start of RT delivery compared to the end. In some sessions, the requirement of the CTV_GTV_ coverage was not met, highlighting the importance of post-treatment CBCT evaluation and the need for further optimization of oART to consistently meet these requirements. The non-ART workflow resulted in statistically higher target coverage for the pelvic lymph nodes, which might be due to the bone match performed during position verification. This could be explained by the limited motion of the lymph nodes relative to the bones [20]. On the contrary, in the oART workflow, the GTV coverage was prioritized over the target coverage of the pelvic lymph nodes during the couch shift. This is a typical challenge for SIB and might be mitigated by opting for a sequential boost strategy.

## Conclusions

RT was challenging for this bladder cancer patient due to the variable bladder filling and the tumor position in the cranial part of the bladder being close to the small bowel. Our case report showed that by treating the patient with oART, the small bowel, and other healthy tissue would have received substantially less dose than if the patient had been treated with non-ART while still having adequate target coverage to the bladder and GTV. Together with the patient showing no long-term toxicity effects during the two-year follow-up, it illustrates the potential superiority of the oART workflow, although larger trials are necessary to confirm these findings.

## References

[1] Shabsigh A, Korets R, Vora KC (2009). Defining early morbidity of radical cystectomy for patients with bladder cancer using a standardized reporting methodology. Eur Urol.

[2] Lutkenhaus LJ, van Os RM, Bel A, Hulshof MC (2016). Clinical results of conformal versus intensity-modulated radiotherapy using a focal simultaneous boost for muscle-invasive bladder cancer in elderly or medically unfit patients. Radiat Oncol.

[3] Zlotta AR, Ballas LK, Niemierko A (2023). Radical cystectomy versus trimodality therapy for muscle-invasive bladder cancer: a multi-institutional propensity score matched and weighted analysis. Lancet Oncol.

[4] Wilson C, Moseshvili E, Tacey M (2020). Assessment of intrafraction motion of the urinary bladder using magnetic resonance imaging (cineMRI). Clin Oncol (R Coll Radiol).

[5] Kong V, Hansen VN, Hafeez S (2021). Image-guided adaptive radiotherapy for bladder cancer. Clin Oncol (R Coll Radiol).

[6] Piet AH, Hulshof MC, Pieters BR, Pos FJ, de Reijke TM, Koning CC (2008). Clinical results of a concomitant boost radiotherapy technique for muscle-invasive bladder cancer. Strahlenther Onkol.

[7] Dees-Ribbers HM, Betgen A, Pos FJ, Witteveen T, Remeijer P, van Herk M (2014). Inter- and intra-fractional bladder motion during radiotherapy for bladder cancer: a comparison of full and empty bladders. Radiother Oncol.

[8] Biancia CD, Yorke E, Kollmeier MA (2014). Image guided radiation therapy for bladder cancer: assessment of bladder motion using implanted fiducial markers. Pract Radiat Oncol.

[9] Stanley DN, Harms J, Pogue JA (2023). A roadmap for implementation of kV-CBCT online adaptive radiation therapy and initial first year experiences. J Appl Clin Med Phys.

[10] Åström LM, Behrens CP, Calmels L (2022). Online adaptive radiotherapy of urinary bladder cancer with full re-optimization to the anatomy of the day: initial experience and dosimetric benefits. Radiother Oncol.

[11] Sibolt P, Andersson LM, Calmels L, Sjöström D, Bjelkengren U, Geertsen P, Behrens CF (2021). Clinical implementation of artificial intelligence-driven cone-beam computed tomography-guided online adaptive radiotherapy in the pelvic region. Phys Imaging Radiat Oncol.

[12] Azzarouali S, Goudschaal K, Visser J (2023). Online adaptive radiotherapy for bladder cancer using a simultaneous integrated boost and fiducial markers. Radiat Oncol.

[13] Foroudi F, Pham D, Bressel M, Gill S, Kron T (2013). Intrafraction bladder motion in radiation therapy estimated from pretreatment and posttreatment volumetric imaging. Int J Radiat Oncol Biol Phys.

[14] Amestoy F, Roubaud G, Antoine M (2019). Review of hypo-fractionated radiotherapy for localized muscle invasive bladder cancer. Crit Rev Oncol Hematol.

[15] Baumann BC, Laugeman E, Kohlmyer S (2023). ARTIA-Bladder: daily online adaptive short-course radiation therapy (RT) and concurrent chemotherapy for muscle-invasive bladder cancer (MIBC): a prospective trial of an individualized approach for reducing bowel and bladder toxicity. Int J Radiat Oncol.

[16] Hunt A, Hanson I, Dunlop A (2020). Feasibility of magnetic resonance guided radiotherapy for the treatment of bladder cancer. Clin Transl Radiat Oncol.

[17] Mitchell A, Ingle M, Smith G (2022). Feasibility of tumour-focused adaptive radiotherapy for bladder cancer on the MR-linac. Clin Transl Radiat Oncol.

[18] den Boer D, den Hartogh MD, Kotte AN (2021). Comparison of Library of Plans with two daily adaptive strategies for whole bladder radiotherapy. Phys Imaging Radiat Oncol.

[19] Foroudi F, Wilson L, Bressel M (2012). A dosimetric comparison of 3D conformal vs intensity modulated vs volumetric arc radiation therapy for muscle invasive bladder cancer. Radiat Oncol.

[20] Hsu A, Pawlicki T, Luxton G, Hara W, King CR (2007). A study of image-guided intensity-modulated radiotherapy with fiducials for localized prostate cancer including pelvic lymph nodes. Int J Radiat Oncol Biol Phys.

